# TNF-α inhibitor induced pigmented purpuric dermatoses: a case report

**DOI:** 10.1186/s41927-022-00255-1

**Published:** 2022-04-29

**Authors:** Divita Jhaveri, Frances Zhao

**Affiliations:** 1 Rheumatology Advanced Trainee, Princess Alexandra Hospital, Brisbane, Australia; 2 Internal Medicine Registrar, Princess Alexandra Hospital, Brisbane, Australia

**Keywords:** Pigmented purpuric dermatosis, TNF-α inhibitors, Adalimumab, Petechiae, Rash, Spondylarthritis

## Abstract

**Background:**

We present a rare case of TNF-α inhibitor induced pigmented purpuric dermatoses (PPD) and explore its mechanisms and management.

**Case presentation:**

A 44-year-old woman presented with non-pruritic non-tender petechial rash on bilateral lower limbs after being started on Adalimumab, with the rash progressing to worsen on Golimumab, both used for managing her seronegative peripheral arthritis. Laboratory panel revealed a negative vasculitis screen and skin biopsy confirmed the condition. After ceasing the TNF-α inhibitors and changing to Secukinumab, an Interleukin-17 inhibitor, the lesions stopped erupting and slowly resolved.

**Conclusion:**

PPD is a benign skin condition and has been associated with various medications and exposure to chemicals in the literature. Different mechanisms have been proposed in the literature however its exact aetiology is unknown. To date, there is no standardized treatment however patients should be reassured that PPD is benign and will often regress by itself once the causative agent has been removed.

## Background

Pigmented purpuric dermatoses (PPD) is a benign skin condition associated with medical conditions such as diabetes, autoimmune conditions and some medications including beta blockers, diuretics and aspirin in the literature. We report a rare case of TNF-alpha inhibitor induced PPD in a 44-year-old woman who was initiated on TNF-alpha inhibitor for treatment of her peripheral arthritis. PPD was diagnosed on skin biopsy and resolved with the cessation of TNF-alpha inhibitors.

The exact aetiology of PPD is unclear at present and no standardised treatment exists to date. However, most studies have found treatment to be of limited value and cessation of the offending agent will eventually lead to clearing of lesions [1, 2], such as in this case.

## Case presentation

A 44-year-old woman of Afghanistani ancestry presented to our rheumatology outpatient clinic with a non-pruritic petechial rash that was symmetrically distributed across both legs. She had a decade long history of peripheral arthritis diagnosed as HLA-B27 positive peripheral spondylarthritis. Her medical history included hypertension, migraine and hepatitis B core antibody positivity with negative HBV DNA on serial bloods.

**Fig. 1:**
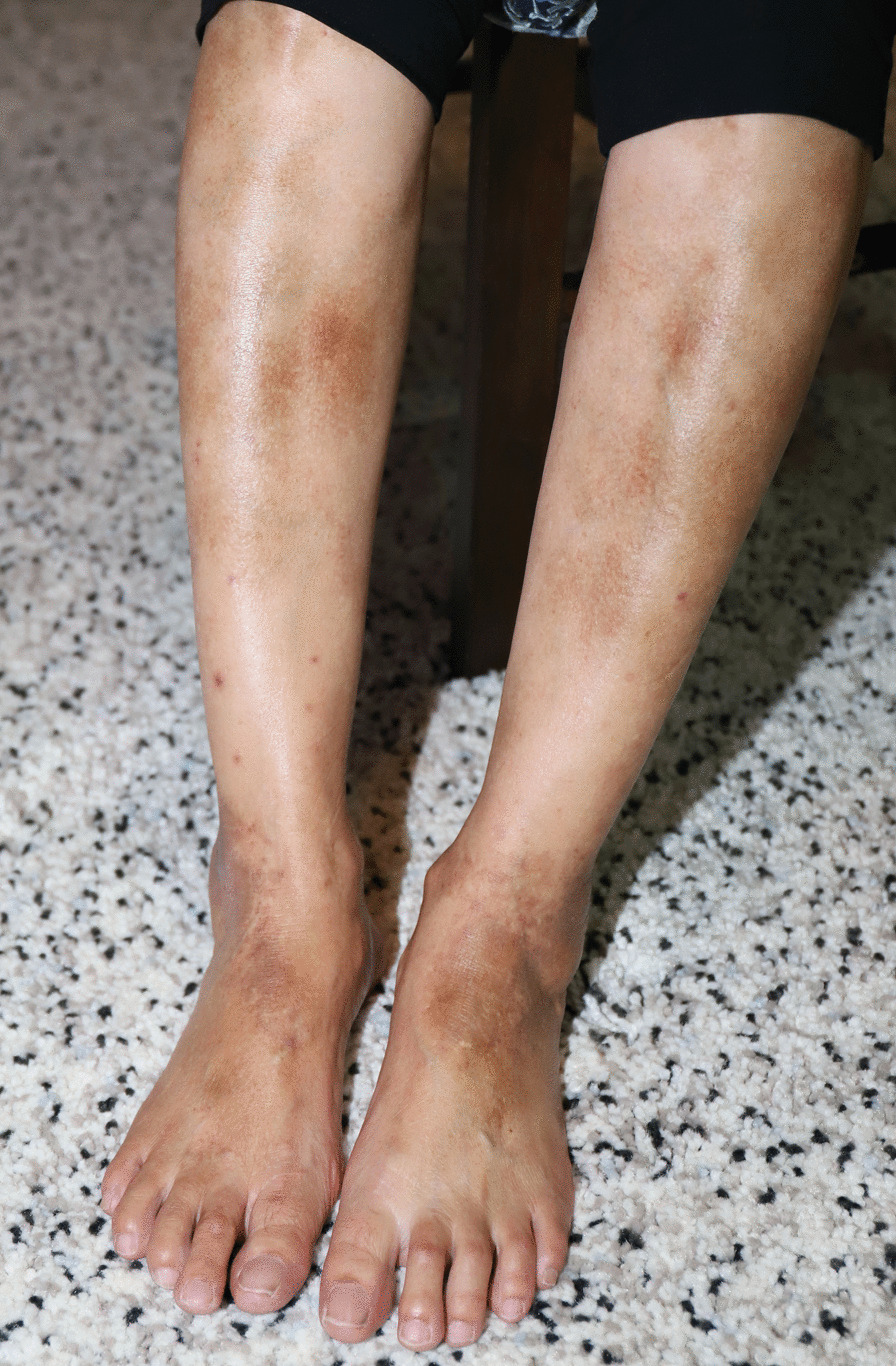
Purpuric dermatoses on patient's lower limbs

**Fig. 2:**
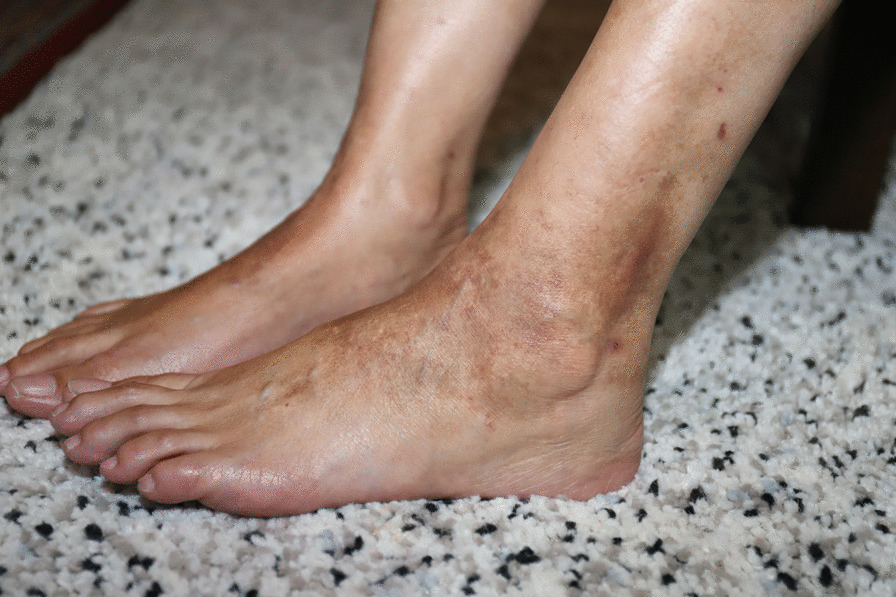
Purpuric dermatoses on patient's lower limbs

Her arthritis was initially treated with sulfasalazine at 500 mg once daily but was ceased due to persistent headaches and nausea and switched to methotrexate at 20 mg weekly. Due to inadequate disease control, the decision was made to escalate to Adalimumab with the cessation of methotrexate and sulfasalazine. Two weeks after its commencement she developed a symmetrical petechial rash on her lower limbs with brown pigmented patches (Figs. 1, 2). During this time, she also developed neutropenia and based on this a decision was made to switch to Golimumab, another TNF-α inhibitor, 3 months after Adalimumab was started. Both agents controlled her peripheral disease. The neutropenia resolved on Golimumab however the rash continued to worsen and progress up to her lower abdomen.

Physical exam was unremarkable with no hepato-splenomegaly or lymphadenopathy. She had no features of psoriasis, uveitis or inflammatory bowel disease on history and examination.

**Fig. 3:**
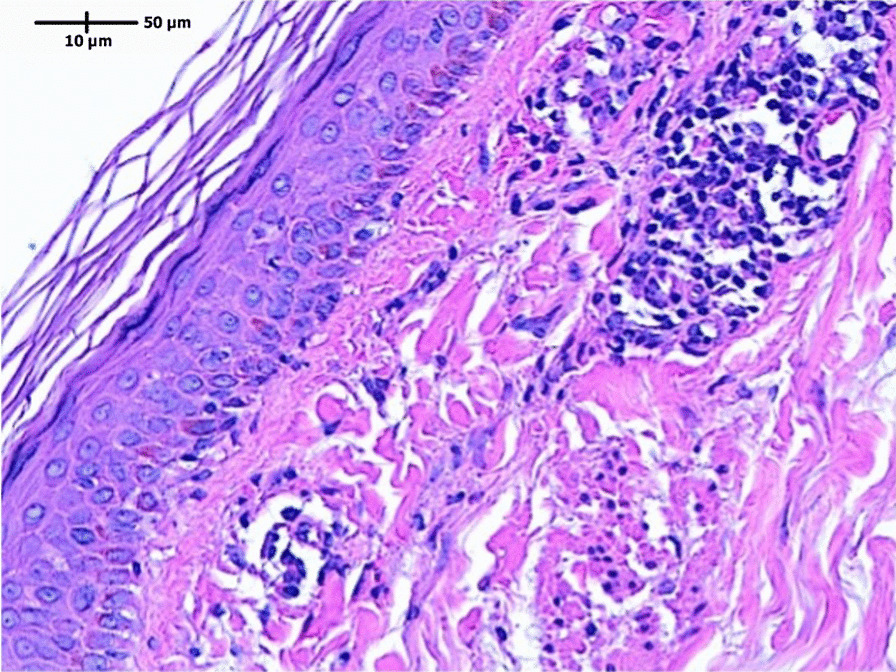
Basal vacuolar change with superficial perivascular lymphocytic infiltrate and presence of red blood cell extravasation

Laboratory testing revealed that her hemoglobin (Hb) was 123 g/L (RR 120–155 g/L); white cell count (WCC) was 4.8 × 10^9^/L (RR 4.5 to 11.0 × 10^9^/L); C-reactive protein (CRP) was 5.0 mg/L (RR < 5.0 mg/L); erythrocyte sedimentation rate (ESR) was 10 mm/1st hour (RR 1–20 mm/1st hour); Creatinine (Cr) was 67 μmol/L (RR 45–90 μmol/L). Vasculitis panel revealed no abnormalities in anti-neutrophil cytoplasmic antibodies (ANCA), serum electrophoresis, urine microscopy and protein. A biopsy of the rash demonstrated basal vacuolar change with superficial perivascular lymphocytic infiltrate and presence of red blood cell extravasation (Fig. 3). PERL’s stain was negative for hemosiderin and PAS stain negative for fungal organisms. The findings were consistent with PPD. Based on the clinical and histopathological findings, it is most likely that this patient had Schamberg's disease, the most common form of PPD.

Golimumab was ceased and she was commenced on Secukinumab, an interleukin-17 inhibitor and was advised that her rash did not require additional treatment. After discontinuing Golimumab, the eruptions stopped within a week and the lesions on her abdomen and legs were noted to have improved significantly 3 months later at her next outpatient review and eventually resolved over the next 6 months.

## Discussion and conclusions

Pigmented purpuric dermatosis (PPD) represent a group of benign conditions, mostly running a chronic relapsing course [1] and it is characterised by multiple petechiae on hyperpigmented macules of yellow to brown colour mostly occurring in the lower extremities [3]. The mechanism of drug induced PPD is unknown but cell mediated immune response appears to play a role [4–7]. The perivascular inflammatory infiltrate has been found to consist of CD4+ T cells [7] (with reduced CD7 expression [6] and CD1a+ dendritic cells [5]. It is hypothesized that the function of cells responsible for structural integrity of the capillaries is altered, leading to extravasation of red blood cells and activates binding of T cells to endothelial cells, fibroblasts, and keratinocytes, via the expression of adhesion molecules [4].

It has been associated with a variety of conditions in the literature including infections, venous hypertension, diabetes mellitus, autoimmune diseases, hematologic disease, dyslipidaemia [3, 8, 9]. Exposure to certain medications have also been reported with the most common being statins, beta and calcium channel blockers, aspirin, diuretics [1].

To our knowledge, this is the second case of TNF-α inhibitor induced PPD reported in literature with the previous being published in the Journal of Rheumatic Diseases (Korea) [10]. The causation mechanisms behind this phenomenon is unclear however reference can be sought from the paradoxical pro-inflammatory effects of TNF-α inhibitor in the treatment of inflammatory bowel disease, ankylosing spondylitis, and rheumatoid arthritis where skin eruptions have been reported as side effects [11]. This could be associated with an increased T-helper type 1 (Th1) and T-helper type 17 (Th17) response in the setting of TNF-α inhibitor, with associations demonstrated in both in vitro animal [12] and human model [13, 14].

It is important to distinguish between PPD and mimics such as vasculitis, venous stasis purpura and thrombocytopenic purpura. Skin biopsy is crucial in the diagnosis of PPD as it is often difficult to distinguish clinically. Leukocytoclastic vasculitis often presents with dermatologic manifestations including purpura and petechiae and presence of systemic symptoms gives clues to the diagnosis of systemic vasculitides such as IgA vasculitis. Another differential for PPD could be venous stasis purpura which are usually co-existent with signs of venous insufficiency such venous ulcers, lower limb edema and varicose veins. It is also important to exclude thrombocytopenic purpura which is associated with thrombocytopenia, specifically a platelet count of < 100–150,000/μL.

To date, no standardized treatment for PPD exists. If it is medication related, spontaneous improvement after discontinuation of the causative drug is said to be common [1, 2]. Small case series and case reports have described clinical response to variety of medications, including topical corticosteroids, oral vitamin C and complementary medicine, however none of them are backed up by large clinical trials to be considered as universal treatment. Studies have found that treatment was of limited benefit and a significant proportion of patients who had follow up data eventually have clearing of lesions.

Pigmented purpuric dermatosis is a group of benign skin eruptions that are characterised by petechial patches and red to purple macules most commonly occurring in the lower extremities. The exact mechanism of pigmented purpuric dermatosis is unknown. Exposure to various medications have been implicated in many cases, although the use of TNF-a inhibitors such as in this case has never been previously reported in the literature. Careful history should be taken to screen for drug induced PPD as a cause. Biopsy can help differentiate this benign condition from its more sinister mimics such as vasculitis. Patients should be assured that this is a benign condition and no treatment is required.

## Methods

Microscopy image captured by LAS X using Leica Microscope.

## Data Availability

Not applicable.

## References

[1] Tolaymat L, Hall MR. Pigmented purpuric dermatitis. StatPearls Publishing; 2021 [updated 2021 July 26]. Available from Pigmented Purpuric Dermatitis—StatPearls—NCBI Bookshelf (nih.gov).

[2] Sharma L, Gupta S (2012). Clinicoepidemiological study of pigmented purpuric dermatoses. Indian Dermatol Online J.

[3] Kim DH, Seo SH, Ahn HH, Kye YC, Choi JE (2015). Characteristics and clinical manifestations of pigmented purpuric dermatosis. Ann Dermatol.

[4] von den Driesch P, Simon M (1994). Cellular adhesion antigen modulation in purpura pigmentosa chronica. J Am Acad Dermatol.

[5] Ghersetich I, Lotti T, Bacci S, Comacchi C, Campanile G, Romagnoli P (1995). Cell infiltrate in progressive pigmented purpura (Schamberg's disease): immunophenotype, adhesion receptors, and intercellular relationships. Int J Dermatol.

[6] Magro CM, Schaefer JT, Crowson AN, Li J, Morrison C (2007). Pigmented purpuric dermatosis: classification by phenotypic and molecular profiles. Am J Clin Pathol.

[7] Smoller BR, Kamel OW (1991). Pigmented purpuric eruptions: immunopathologic studies supportive of a common immunophenotype. J Cutan Pathol.

[8] Sardana K, Sarkar R, Sehgal VN (2004). Pigmented purpuric dermatoses: an overview. Int J Dermatol.

[9] Tristani-Firouzi P, Meadows KP, Vanderhooft S (2001). Pigmented purpuric eruptions of childhood: a series of cases and review of literature. Pediatr Dermatol.

[10] Moon SH, Ko JY (2014). Dermatological side effects of anti-tumor necrosis factor alpha therapy. J Rheum Dis.

[11] Fiorino G, Danese S, Pariente B, Allez M (2014). Paradoxical immune-mediated inflammation in inflammatory bowel disease patients receiving anti-TNF-α agents. Autoimmun Rev.

[12] Notley CA, Inglis JJ, Alzabin S, McCann FE, McNamee KE, Williams RO (2008). Blockade of tumor necrosis factor in collagen-induced arthritis reveals a novel immunoregulatory pathway for Th1 and Th17 cells. J Exp Med.

[13] Hull DN, Williams RO, Pathan E, Alzabin S, Abraham S, Taylor PC (2015). Anti-tumour necrosis factor treatment increases circulating T helper type 17 cells similarly in different types of inflammatory arthritis. Clin Exp Immunol.

[14] Alzabin S, Abraham SM, Taher TE, Palfreeman A, Hull D, McNamee K (2012). Incomplete response of inflammatory arthritis to TNFα blockade is associated with the Th17 pathway. Ann Rheum Dis.

